# Efficacy of the laser at low intensity on primary burning oral syndrome: a systematic review

**DOI:** 10.4317/medoral.24144

**Published:** 2021-02-20

**Authors:** Ana Liz Pereira de Matos, Pedro Urquiza Jayme Silva, Luiz Renato Paranhos, Ingrede Tatiane Serafim Santana, Felipe Rodrigues de Matos

**Affiliations:** 1 Postgraduate Program in Sciences Applied to Health. Federal University of Sergipe, Lagarto / SE, Brazil; 2 Department of oral and maxillofacial pathology. Federal University of Uberlândia / MG, Brazil; 3 Department of Social and Preventive Dentistry. Federal University of Uberlândia / MG, Brazil; 4 Postgraduate Program in Physiological Sciences. Federal University of Sergipe, São Cristóvão / SE, Brazil; 5 Department of Dentistry. Federal University of Sergipe, Lagarto / SE, Brazil

## Abstract

**Background:**

Primary burning mouth syndrome (BMS) is a chronic clinical condition of idiopathic mainly characterized by pain and a burning sensation in the oral cavity. The application of laser at low intensity therapy is a treatment option. This systematic review evaluated the efficacy of laser therapy in treating symptoms of burning mouth syndrome.

**Material and Methods:**

The study was formulated according to the PRISMA and Cochrane guidelines. Seven databases were used as primary sources of research. Only randomized controlled clinical trials were included. The efficacy of the therapy was estimated comparing the values of the visual and numerical scales of pain before and after laser treatment, through qualitative analysis.

**Results:**

The search resulted in 348 records and only eight filled the eligibility criteria and were included. All studies evaluated pain and / or a burning sensation considering a time interval of two to ten weeks. The total sample consisted of 314 patients submitted to treatment: 123 from the control group, who participated with laser off or with the tip blocked, and 191 from the intervention group, treated with low-level laser therapy. The female gender stood out and the average age of the participants was 60.89 years. The main symptoms reported were pain and a burning sensation in the oral mucosa and tongue. The parameters adopted by the authors for laser treatment were diverse and the variables were not fully described in the published studies. Visual analog and numerical scales were used to assess symptoms and only three studies showed statistical significance.

**Conclusions:**

It is suggested that laser therapy may be an effective alternative in the treatment of BMS. New randomized clinical trials should consider well-established protocols to better understand the efficacy of laser therapy without confounding the effects.

** Key words:**Efficacy, systematic review, burning mouth syndrome, low level laser therapy.

## Introduction

The Burning Mouth Syndrome (BMS) is mainly a chronic medical condition, idiopathic characterized by pain with burning sensation in the oral cavity without any dysfunction and apparent or detecTable organic cause (1). The epidemiology of BMS is not fully known, having a high occurrence in postmenopausal women (2,3). The general prevalence is still unreliable due to the large variation in published studies (0.01% to 40%), with a tendency to increase with advancing age (4).

BMS has a complex pathogenesis involving psychogenic factors and deregulated peripheral and central pain, not existing a standard treatment protocol for management (5). The diagnosis of all symptoms in each patient of BMS must be analyzed meticulously and carefully. It is diagnosed by exclusion and it has among others, topical and systemic treatments, seeking the decrease of the symptoms (6).

The low intensity laser therapy (or biomodulation) is an alternative for the treatment of symptoms of BMS. The low intensity laser (LLLT) is the application of light with a low power laser or LED that promotes tissue regeneration, reduces inflammation and relieves pain (7). There is no thermal effects and analgesia in LLLT is usually gradual, cumulative and requires multiple sessions, besides not having side effects (8).

The mechanisms of therapeutic effect of low-level laser therapy occur through a variety of processes which pass through a wavelength of incident light, monochromatic and polarized, the absorbed photons activate and trigger specific biochemical or physiological responses and the entire sequence of events starts with photon absorption and ends with systemic effects (9).

Researchers defend the primary role of low intensity light in mitochondria (8,10). When mitochondria meets the light, the cytochrome c oxidase increases the production of adenosine triphosphate (ATP) by phosphorylation oxidative. In addition, it modulates reactive oxygen species and induces transcription factors (8). These effects, in turn, lead to cell proliferation and migration (particularly by fibroblasts), also lead to modulation in cytokine levels, growth factors, inflammatory mediators and increases tissue oxygenation (10).

Until then, the literature presents controversial results on the effectiveness of low-level laser therapy in Burning Mouth Syndrome, by comparing the reduction of pain symptoms and burning sensation. Clinical trials have brought beneficial results with reduced pain and burning sensation in patients with BMS treated with LLLT (11-13), while another trial concluded that both LLLT and placebo reduced the symptoms of BMS (14).

There is evidence that points to the influence of local inflammatory processes in the oral cavity and systemic in association with the pathophysiology of BMS. Inflamed tissues produce more reactive oxygen species - a by-product of inflammation, which compromises the production of ATP in cells. The production of nitric oxide (NO) in the mitochondria in cases of injury, such as inflammation, can inhibit as mitochondrial airways, as NO is reversibly bound to cytochrome c oxidase, an essential enzyme in this airway. The low intensity laser, in turn, can reverse this bond between NO and cytochrome c oxidase by changing the redox potential of the cell, allowing it to produce ATP and accelerate the process of repairing the inflammatory reaction, improving the symptomatic condition of BMS (15).

Before the exposed controversy, the aim of this study is to evaluate the effectiveness of therapy of low-level laser therapy in the treatment of symptoms of Burning Mouth Syndrome primary through a systematic review of current literature.

## Material and Methods

-  Protocol and registration

This systematic review was carried out in accordance with the recommendations of PRISMA (preferred reporting items for systematic analyzes and meta-analyzes) (16) and the Cochrane guidelines (17). The systematic review protocol was registered in the PROSPERO database (CRD42021226064).

-  Study design and eligibility criteria

This study was a systematic review based on the PICO strategy, in order to answer the following question: “Is low-level laser therapy effective in reducing the symptoms of Burning Mouth Syndrome (BMS)?" For this purpose, the population was patients with primary Burning Mouth Syndrome; intervention - low intensity laser therapy; Patients were compared with Primary Burning Mouth Syndrome treated with Laser low intensity therapy versus placebo - and as a conclusion, the patients’ pain and a burning sensation in the oral cavity decreased.

The inclusion criteria were randomized clinical trials, with no period restriction, published in any language, which dealt with the evaluation of the effectiveness of low-level laser therapy in the treatment of the symptoms of Burning Mouth Syndrome.

The exclusion criteria were studies outside the scope of this systematic review, *in vitro* studies, performed on animals, case reports, letter to the editor and / or editorials, literature review, books and book chapters, pilot study and indexes and abstracts or university work assignment with insufficient data (letters, personal opinions, conference abstracts).

- Sources of information and research

All steps were taken to minimize the biases of selection and publication. The base data PubMed (including Medline), Scopus, Embase, SciELO, Web of Science, Latin-American and Caribbean Health Sciences (LILACS) and Cochrane were used as primary sources of study. The OpenGrey and Open Access (OATD) theses and dissertations were used to access the "gray literature" to avoid bias in relation to the lack of published negative results.

The resources MeSH (Medical Subject Headings), DeCS (Health Sciences Descriptors) and Emtree (Embase Subject Subject Headings) were used to select the search descriptors. The Boolean operators “AND” and “OR” were used to improve the search strategy through various combinations.

The first bibliographic search was carried in April 2020. The results obtained were imported into EndNote Web ™ software (Thomson Reuters, Toronto, Canada) to remove duplicates. The rest of the results were imported into Microsoft Word ™ 2010 (Microsoft ™ Ltd, Washington, USA), in which the remaining duplicates were removed manually. 

- Study selection

Two independent reviewers [ALPM and PJU] were previously calibrated on a sample of 20% of the studies and reached an appropriate agreement between the examiners. The eligibility review was carried out independently by these reviewers, with disagreements resolved by discussion with a third reviewer [LRP] to reach consensus.

The selection of studies was carried out in two stages. The first stage included a thorough analysis of the titles and abstracts of the articles. Reviewers were not blind to the names of authors and journals. Studies with titles unrelated to the topic of interest in our review were eliminated at this stage. The titles that met the objectives of our study, but had no abstracts available, were fully analyzed in the second stage.

In the last stage, the eligible studies had their full texts obtained and evaluated to verify whether they met the eligibility criteria. The references of the eligible articles were carefully evaluated to verify studies that were not detected in the main search strategy. The excluded studies were registered separately, together with the reasons for exclusion.

- Data collect

After selection, the studies were analyzed by two reviewers [ALPM and PJU], who extracted the following information from the articles: identification (author, year, country and place of the research), characteristics of the sample (number of patients in each study, sex distribution, average age, laser parameters and pain analogue scale).

- Risk of individual study bias

The Critical Assessment Tools of the Joanna Briggs Institute (JBI) for using in Systematic Reviews for Randomized Clinical Trials (18) were used to assess the risk of bias and the individual quality of the selected studies. Two authors [ALPM and PJU] independently assessed each domain in relation to the potential risk of bias, as recommended by the PRISMA statement (16). Each study was categorized according to the percentage of positive responses to the questions corresponding to the assessment instrument. The risk of bias was considered high when the study obtained 49% of the answers "yes", moderate or medium when the study obtained 50% to 69% of the answers "yes" and low when the study reached more than 70% of the score "yes" ".

- Data analysis

The data collection process was carried out through analysis of the selected studies, and the conclusion was presented in a descriptive / narrative manner, analyzing the heterogeneity of the studies. A meta-analysis was planned if the data from the eligible studies were homogeneous.

## Results

During the first phase of study selection, 348 papers were found. One study was found in the “gray literature”, but it did not meet the objective and was removed manually. After removing duplicates, 203 papers remained for analysis of titles and abstracts. After a detailed analysis, only seven studies were eligible to review the full text.

**Figure 1:**
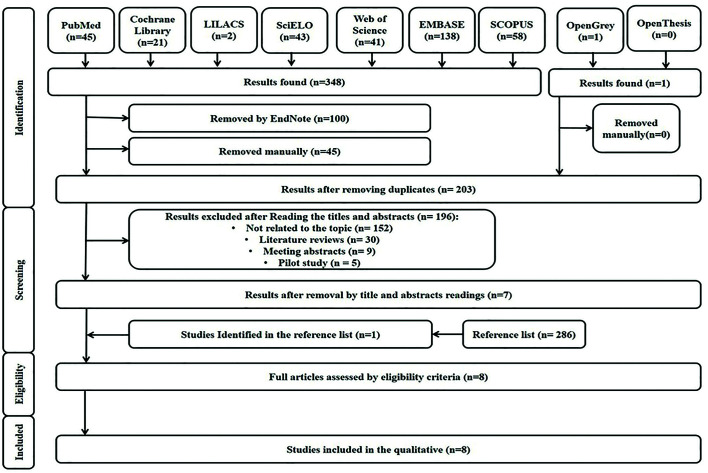
Flowchart of the literature search and selection process adapted from the PRISMA statement.

The references of these eligible studies were carefully evaluated, and an additional article was selected. None of these seven studies were excluded for the purposes of qualitative analysis (review). Fig. 1. reproduces the process of searching, identifying, including and excluding articles.

The studies were published between 2015 and 2020 and carried out in Spain (13,19,20), Brazil (12,14), Italy (21), Croatia (22) and Iran (11). The sources of information on the demographic and clinical characteristics of the population are available in Table 1.

All articles were approved by the Ethics Committee of the respective institution or hospital and reported that informed consent was obtained before the study started. Only two studies followed the CONSORT statement (11,19). Five of the eight eligible studies reported decrease in symptoms after application of therapy to laser. Two studies presented the registration number of their randomized controlled trial (11,19).

Two studies suggested the need for further research with established protocols and larger samples to determine the real effectiveness of laser therapy in BMS (21,22). Pedro *et al*. [2020] (19) pointed out the need furthermore also with longer follow - up and more sessions to assess the possibility of periodic application of therapy to laser.

The groups were allocated to part of the sample "group laser - irradiation received by infrared laser or red" and "control- group received no irradiation," the laser has been turned off or has been blocked end. Only one study (12) used red laser and two other groups with the same infrared laser in equal parameters, but with differences in treatment time.

**Table 1:**
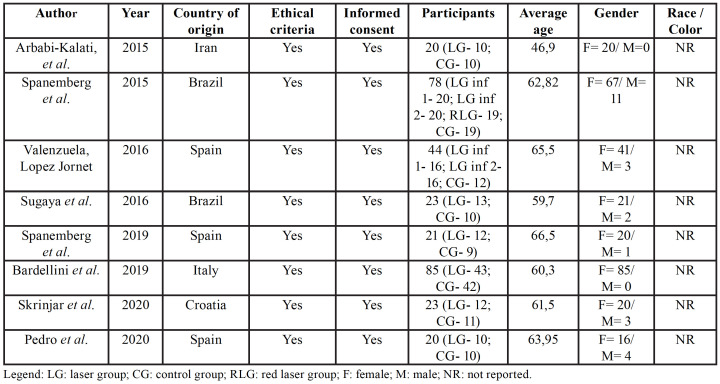
General characteristics of the studies.

The study by Valenzuela and Lopez Jornet [2016] (13) used two groups with the infrared laser, but in different parameters. Table 2 summarizes the main characteristics of the eligible studies regarding the parameters used for therapy the laser.

As for the risk of bias, two studies were high risk and four studies were medium risk. Only two studies (19,21) had a low risk of bias. Table 3 presents these detailed results, assessed by the Joanna Briggs Institute (JBI) Critical Assessment Tools for use in Systematic Reviews for studies of randomized controlled trials (18).

**Table 2:**
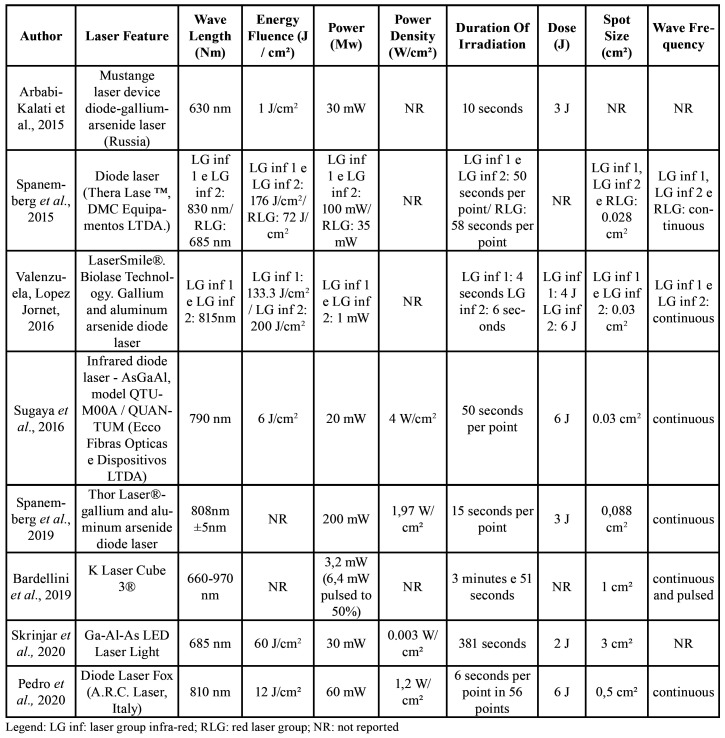
Summary of the main characteristics of the eligible studies.

**Table 3:**
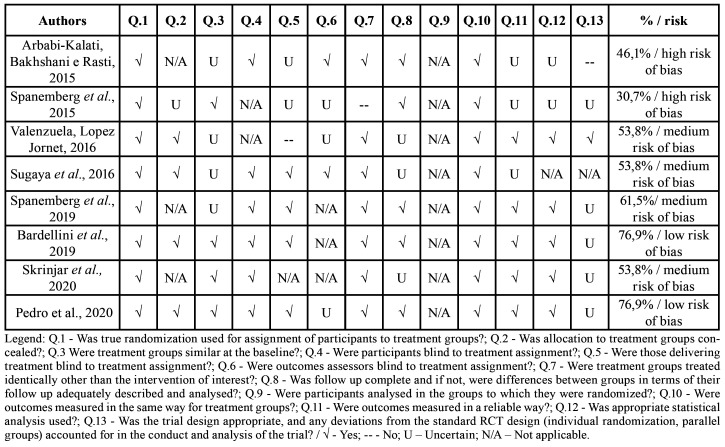
Risk of bias assessed by the Joanna Briggs Institute Critical Assessment Tools for use in JBI Systematic Reviews for studies of randomized clinical trials.

Most studies used the Visual Analogical Scale (VAS) to assess pain and burning sensation in the participants. Arbabi-Kalati , Bakhshani and Rasti [2015] (11) used a numerical display scale (EVN) and Spanemberg *et al*. [2015] (12) used VAS and EVN. Seven studies considered scales with values ​​from zero to ten (zero refers to no pain or burning sensation and ten intense pain and / or burning). Except Sugaya *et al*. [2016] (14) who considered zero to five. Table 4 shows the average VAS and EVN scores contained in the studies, in addition to the time of follow-up of treatment and their outcome.

Sugaya *et al*. [2016] (14) presented the VAS values on average differently from other studies. They individually reported the participants by received sessions and categorized the values ​​by percentage. It was considered: 0- no burning sensation (0%); 1- excellent reduction in the burning sensation (1–25%); 2- good decrease in the burning sensation (26-50%); 3- regular decrease in the burning sensation (51-75%); 4- burning sensation unchanged (76-100%); 5- the burning sensation worsened (> 100%). The control group started the first session with an average of 52%, in the regular category. In the fourth session, it presented 31% - a good burning sensation. The intervention group, in turn, had an average of 49% in its first session - good burning sensation and ended with 18% - excellent burning sensation.

Bardellini *et al*. [2019] (21), presented the values ​​of statistical significance, without presenting the average values ​​of VAS in a Table. According to the authors, before the sessions, the EVA score was similar in both groups (*p*= 0.75). After the 5th fifth session, there was a reduction in the average VAS, but without a statistically significant difference between groups (*p*= 0.6232). At the end of therapy, patients treated with LLLT showed a significant decrease in symptoms (*p*= 0.0008), maintained at follow-up the day after the last session (*p*= 0.0005).

The places of application of laser therapy varied in the studies. All irradiated areas where the participants reported the symptoms of pain and burning sensation. Four studies did not report the exact locations of application (12,14,21,22). Two studies (13,14) reported that the application was on the oral mucosa, without describing the locations.

Arbabi-Kalati , Bakhshani and Rasti [2015] (11) irradiated ten areas of the oral mucosa - two areas on the oral mucosa on each side, two on the tongue, two on the floor of the mouth, one area on the soft palate and one on the hard palate. Spanemberg *et al*. [2015] (12) illuminated seventeen points on the tongue (three on the apex, four on the side and ten on the back), eight points on the oral mucosa, five on the lip mucosa, eight points on the hard palate, three points on the soft palate and gums.

Spanemberg *et al*. [2019] (20) used fourteen points on the tongue (three on the tip, four on the lateral edge, ten on the dorsal surface), eight points on the oral mucosa, five on the lip mucosa, eight on the hard palate, three on the soft palate and three points on the gum. Pedro *et al*. [2020] (19) irradiated four quadrants of the buccal mucosa - four on each lip mucosa, six points on each of the two buccal mucosa, six on the hard palate, four on each lateral edge of the tongue, six on the back of the tongue and four sublingual points.

**Table 4:**
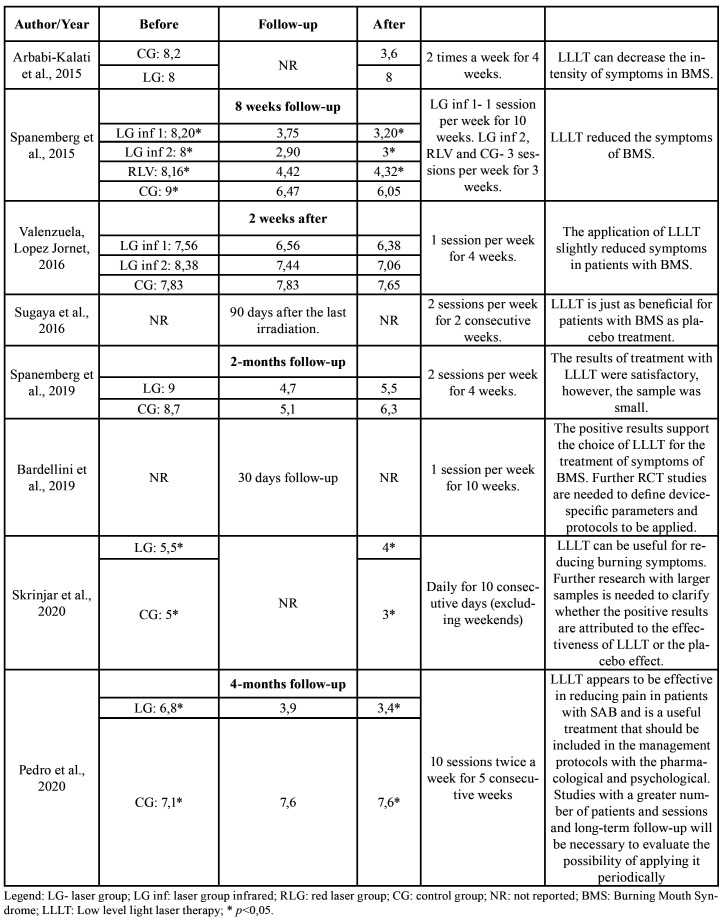
Average comparison of pain scales before and after laser therapy with the outcome.

## Discussion

The therapy low laser intensity is still considered a new alternative in need of greater understanding of its mechanism of action through high-quality studies with larger sample sizes and longer periods of follow-up (23), although there are controversies about the real effectiveness of this therapy.

The sample of this review was variable regarding the number of participants. The majority was women with average age that corresponds to the post-menopausal age group. As it affects women, at this stage of life, who may have hormonal instability, the hypothesis of hormonal participation as a cause of BMS is supported. This can be considered a triggering factor in the development and progression of the pathology, but the definitive relationship between BMS and hormonal changes is not yet established (24).

Regarding the wavelength, the red and infra-red spectra (between 390 to 1100 nm) offer stimulating interactions with the biological tissue (15). The studies in this review used wavelengths ranging from 630 to 970 nm. The lower wavelengths induce stimulating effects, while the higher levels provide inhibitory effects (23). A small stimulus of the therapy LLLT may not have a biological effect, while large stimulus may cause inhibitory or cytotoxic effects, by the production of excessive reactive oxygen species excessive that can inhibit production of mitochondrial energy and generate apoptosis (25).

In LLLT, it is not allowed to illuminate the same area with different wavelengths due to the inhibitory action (9). Bardellini *et al*. [2019] used different wavelengths and frequencies in their applications, which can generate interferences and inhibitory effects (21).

Energy (J) and dose or fluency (J / cm2) often not reported in studies, however, the absence of this information does not prevent the guarantee of an effective laser therapy (9). The laser dose is not shown as a single parameter, but as a set of physical parameters, including power density, creep, radiated effective area, beam intensity profile, duration of exposure, length of length wave or wavelength distribution, total number of exposures and the time between exposures and total fluency (9).

Among the parameters mentioned above, a systematic review pointed out that the fluency or dose of energy should be in the range of 0.5 to 8 J / cm 2 because it can reduce inflammation and accelerate wound healing (26). Most of the studies analyzed, in this review, used energy fluency higher than recommended by the literature. Two studies far exceeded these values: In Spanemberg *et al*. [2015] (12), Valenzuela and Lopez- Jornet [2016] (13), no differences were observed before and after treatment on the VAS scale.

Six of the eight studies analyzed used continuous waves in the treatment (12-14,19-21). The laser of continuous wave may provide unwanted effects, especially with regard to thermal losses; the lasers super pulsed are capable of acting in a therapeutic manner with deep penetration and shorter periods of treatment, without undesirable these effects (15).

Pulsed lasers provide additional benefits related to pulse attenuation intervals after activation times and require less tissue heating (15). Although the literature theoretically discusses this information, in practice, the continuous wave can work without causing heating in the tissues, since studies of this sample have shown improvement in the symptoms of pain and burning through the average values ​​of the VAS scale.

The time of application is the same for different cell types and is between 100 and 300 seconds (27). Most of the studies in this review did not detail the total exposure time of the tissue to irradiation. Instead, they showed time irradiated by point. One of the two studies that reported the total time exceeded the recommended: they radiated for 381 seconds (22). One area should not be irradiated for more than five minutes and the total treatment time (including all areas) should not exceed twenty minutes (9).

LLLT has an analgesic effect; however, the application must be successive, continuous and in several sessions (8). Six of the eight studies carried out 8 to 10 sessions. Two studies followed the therapy with only 4 sessions (13,14). Both had little difference in the VAS scale score at the end of treatment. Thus, the importance of longer follow-ups is suggested.

It was observed that only the study by Sugaya *et al*. [2016] (14) reported all the parameters used in the laser application. The others failed to present important data that prevent the in-depth analysis of the functionality of the therapy in relation to the presented outcome.

Seven studies in the sample reported outcomes that point to the effectiveness of low-level laser therapy, with reduced pain and burning sensation in the treated participants; three of them demonstrated statistical significance between the groups (12,19,22).

The therapy the laser low level is described as having minimal side effect and presents a reduction of symptoms in BMS. Some and clinical trials have reported decreased pain and burning sensation in BMS after treatment with LLLT (11-13,19,20). Skrinjar *et al*. [2020] (22) further emphasized that the available results make it impossible to clarify whether the positive results are attributed to the effectiveness of LLLT or the placebo effect.

To understand the role of laser in BMS, the pathophysiology of this condition must be highlighted. There is some evidence pointing to the influence of local inflammatory processes in the oral cavity and systemic in association with the pathophysiology of BMS. According to Barry *et al*. [2018] (28), pro-inflammatory cytokines are generally linked to nociceptive signaling and are elevated in disorders involving neuropathic pain. This study found elevated plasma levels of the pro-inflammatory cytokine IL-8 in patients with BMS, when compared to plasma from healthy volunteers, and this increase correlated with levels of pain in the oral cavity and depressive symptoms (28).

Another study proved the increased and statistically significant concentration of cytokines IL-2 and IL-6 in all saliva samples from participants with BMS, when compared to healthy individuals (29). Ribarić *et al*. [2013] (30) found results that, in addition to demonstrating an increase in pro-inflammatory cytokines TNF-α and IL-6 in participants with BMS, found a significant reduction in the salivary levels of these cytokines after treatment with red LLLT for four weeks. Finally, Treldal *et al*. [2019] (31) tested a local anesthetic lozenge (bupivacaine) on people with BMS, and assessed inflammation in blood plasma and saliva. The group that did not receive the lozenge tended to have high levels of IL-6, IL-8, IL-17, IL-23 and TNF-α in the plasma, compared to the group that was treated.

Taken together, these results show that inflamed tissues produce a greater amount of reactive oxygen species - a by-product of inflammation - that compromise the production of ATP in cells. The production of nitric oxide (NO) in the mitochondria in cases of injury, such as inflammation, can inhibit the mitochondrial airways, as the NO is reversibly bound to cytochrome c oxidase, an essential enzyme in this airway. The low-intensity laser, in turn, can reverse this binding of NO with cytochrome c oxidase by changing the redox potential of the cell, allowing it to produce ATP and accelerate the process of repairing the inflammatory reaction, improving the symptomatic condition from BMS (15).

A limitation of this systematic review, it can be noted that the studies showed heterogeneity in the application protocols therapy to laser; in addition, there were inconsistencies in the report of the average score of the VAS scale, which hindered the in-depth analysis of the results, limiting the meta-analysis of the data.

New well-conducted and standardized clinical trials should be planned using ideal parameters based on the latest available evidence and analysis of the limitations of published clinical trials, with the purpose of controlling bias and better understanding of laser therapy in BMS.

## Conclusions

In this review, could be suggested that laser therapy might be effective in treating an alternative BMS. The sample consisted of women with an average age of 60.89 years, a period that corresponds to post-menopause.

The most frequent symptoms were pain and a burning sensation, felt mainly in the oral mucosa and tongue. The scores on the VAS scale in three studies were lower in the laser- treated group.

BMS is still a clinical condition under investigation and an understanding of its pathophysiology is essential for planning treatments that really work, without confounding effects. Other randomized trials should consider protocols well established laser therapy.
